# The Institutional Worker

**Published:** 1913-02-01

**Authors:** 


### INSTITUTIONAL WORKER

**Being a Special Supplement to "The Hospital"**

### BUREAU OF INFORMATION.

### Rules for Correspondents.

1. Every letter, on whatever subject, must be accompanied by the coupon to be cut from the back cover (inside page) of The Hospital, current issue, and must contain the name and address of the correspondent with pseudonym for publication if desired.

2. Replies by post cannot be given, save under exceptional circumstances at the Editor's discretion.

3. Letters from Approved Homes in reply to special needs published in the Bureau should state terms and full particulars, and be sent prepaid under cover to the Editor of the Bureau with name written across coupon for identification.

4. Proprietors of Homes which have not yet been entered on the List of Approved Homes, but have spare accommodation likely to suit special needs, are invited to write for an application form for registration. The fee for registration, which includes two announcements of the Home in the Bureau, is 5s.

5. All communications to be addressed to the Editor of The Hospital, 28 Southampton Street, Strand, London, W.C., and marked "Bureau of Information."

**INVITATION TO READERS.—The Editor is prepared to forward to applicants, without fee on either side, offers of accommodation from Homes on the Approved List suitable for medical and surgical cases, for the aged, for chronic or mental patients, for electrical treatment and massage, for convalescents and for children, at varying terms, in any required locality. Reduced terms can often be arranged. The special needs of any who may be sick or in difficulties are also made known in these columns free of charge.**

### Approved Homes Recently Added to List.

Note.—*No Home is added to the List without medical and other testimony to its good management.*

**Boscombe, Hants.**—Riffelberg, 10 Hamilton Road. Principal: Miss L. A. Fisher. Medical and surgical cases, rest-cure, chronic. Attractive central situation on high ground. Electrophone. Tel. 1169.

**London.**—198 Cromwell Road, South Kensington.—Principal: Miss Bartholomew. Thoroughly high-class and fully-equipped Nursing Home, for medical, surgical, and other cases.

### Nursing Homes Wanted.

**For Old Lady.**—The patient is suffering from senile decay. A comfortable Home is wanted at very moderate terms in London or suburbs. Very good references can be given.—Miss McQ. (147a).

### Training and Employment.

**Free Training as Midwife.**—Many of the County Nursing Associations offer free training to midwives from time to time, and as your candidate has some knowledge of nursing she might have a good chance of being chosen. Write to the Secretary of the Nursing Association at the County Hall of the capital town in several counties and ask for particulars. She would have to bind herself to serve for a term of years (three or four) in the county for which she was trained.—Mrs. A. P., Hawkhurst Rectory.

**Consulting Room Nurse.**—We fear you will have considerable difficulty in obtaining the post you require as private nurse in the consulting room of a "well-known physician or surgeon." Usually the nurse who performed this kind of duty would be expected also to answer letters, make appointments, etc. Your best chance is to watch the advertisement columns in medical and nursing papers, and apply with typed copies of testimonials; but we must warn you that these posts are usually filled by private influence, as doctors naturally choose their nurse from those who have worked under them. The salary given would be from £80 a year if the nurse's whole time were engaged.—Sumatra.

**Handbook on Chiropody.**—A very useful little book on "Chiropody: Training and Practice," by H. C. Sexton, can be had from The Scientific Press, 28 and 29 Southampton Street, Strand, London, W.C., for 1s. net. A scientific course of training in chiropody is not easily obtainable in England, but any good private chiropodist would doubtless arrange to give you lessons on condition that you did not compete in that particular locality.—Hygeia.

**Training at Eighteen.**—It is practically impossible to get admission to a training school, either with or without salary, at this age. Mental hospitals take probationers at eighteen, and after a three years' course of mental training you would be ready for hospital work. Let us know if this appeals to you, and we will advise further.—M. E. B., Herne Hill.

### Nursing in the Empire and Abroad.

**Nursing in Carlsruhe.**—Prospects are not particularly alluring, as the ordinary rate of pay for private nurses is from £15 to £30 a year, and the English colony is a small one. If, however, you are accompanying an invalid and wish to take another case afterwards you could write to the British Chaplain at Heidelberg, who would know if your services would be useful in that town. There are more English residents in Freiberg and Baden Baden than at Carlsruhe, and therefore more chance of remunerative work.—Elsa.

**Hospital Accommodation in Cyprus.**—There are six hospitals in the island, all offering accommodation to private patients. The Nicosia Hospital has three trained nurses, but the others mostly depend on native nurses. The supply of nurses is met by the Colonial Nursing Association.—James P.

**Nursing Home in Wiesbaden.**—If you will send us a stamped envelope we will forward you the address of the German physician in Wiesbaden who has informed us of the need for a Nursing Home in this town. You could not form a sound judgment on so important a matter as starting a Home without first paying a preliminary visit and examining into rents, available houses, and nursing prospects generally. The resident population is over 100,000, and visitors number 180,000, and of the latter some thousands are of British nationality. At present there is no Nursing Home, and it is believed that one conducted at moderate terms by a nurse who, like yourself, is thoroughly acquainted with the German life and language, would be immediately successful.—Wanderer.

**Nurses Wanted in Santiago.**—An emphatic answer in the affirmative was returned to the inquiry we have addressed to a competent authority on the spot whether any demand exists for trained nurses in this locality. This capital city of Chile is beautifully situated on a wide plain 1,860 feet above sea-level, below the main range of the Andes.—J. P.

### Miscellaneous.

**Nurses' Association.**—Do you mean a nurses' co-operation or agency for sending out nurses? No doubt if there is a demand in your neighbourhood for nurses and no agency to meet it, you would find it pay very well and fit in with the other side of the work. It is easier to organise a night and day service when nurses are already in the house. You must be guided entirely by the reports of the medical men in the neighbourhood, all of whom should be consulted. Without strong encouragement in this direction the onus of finding regular employment for a staff of nurses should not be attempted. It is outside the scope of the Bureau.—G. M.

### Letter-Box.

**Communications have been received and attended to from:**—Mr. I. R. M., Westbourne Park Square; Miss W., South Woodford; Miss N. E. N., Burgess Hill; Mrs. H. Q., Portishead; Miss E. A. T., South Kensington; Miss M., Wimbledon; Sister A. C. I. G., Isley.

**The Editor begs to acknowledge the receipt of communications with enclosures from:**—Miss Fisher, Nurse Code, Miss MacDonald, Miss Kick, Miss Morris, Miss Bartholomew.

**The Editor is much indebted for testimonials received in response to enquiries from:** Dr. Bartlett, Dr. Bowney, Mrs. Stuart Thompson, Mrs. Holt, Mrs. Becke, Dr. Crofts Neville, E. Hair, Esq., Miss Taylor.

**Letters forwarded from:**—"Charity," Miss G., Nurse McC., Mrs. A., Miss H., Sister A., Misses G. and H., Mrs. D., Miss R., Misses G. and von L., Miss S.

The Editor is unable to introduce patients to any but the Homes on the Approved List.—Miss Mary Pollard; Miss M. Metcalf.

We are sorry that it does not fall within the scope of the Bureau to recommend private nurses.—Miss C. M.; "Holinna."

The advice was intended for heads of Nursing Homes wanting patients, not for nurses seeking posts. We are sorry we cannot help you.—Miss Grace S.

Your best plan will be to study the advertisement columns. We cannot undertake this kind of recommendation.—Mrs. Golby.

Will the lady who kindly wrote to "Charity" *re* adoption of baby communicate with the Editor?

The Editor has received a letter from a lady residing "about ten miles from Dawlish" who wishes to receive a patient. As the letter has no date, no name, no address, and encloses neither stamp nor coupon, it is difficult to understand what she wishes us to do.

### Institution Facts and Figures.

QUESTIONS FOR FEBRUARY.

1. State the quantity of potatoes used in your institution during the month of December, and the price per cwt. paid for them. Enumerate all other kinds of vegetables, cooked or raw, which were served during this month. Was any of this produce grown in the institution gardens? Please state the number of beds.

2. Give the diet for convalescent patients in your institution, and state what it costs per week at the present prices.

The figures must be the result of actual observation and competition, not estimates. Either or both of the questions may be answered by each contributor. The best answers will be published, and for each contribution considered by the Editor to be of sufficient interest for publication payment of FIVE SHILLINGS will be made.

The following rules must be observed by contributors to the FACTS AND FIGURES Column:—

1. Answers must be written on one side of the paper. They must bear the name and address of the sender and be accompanied by coupon to be cut from the back cover (inside page) of the current issue of THE HOSPITAL. A pseudonym must be chosen if the name is not to be published.

2. Answers must be addressed to the Editor of THE HOSPITAL, 28-29 Southampton Street, Strand, London, W.C.; they must be marked in the left-hand corner "Facts and Figures," and must be received before the end of the month.

SELECTED ANSWERS TO JANUARY QUESTIONS.

The answers to the January questions have been very disappointing. Why has no one volunteered to describe the method of making beef-tea in use in their institution? Can it be possible that no official had sufficient knowledge of the prices paid for the meat to be able to work out the price per pint of the liquor? This seems incredible, but otherwise the omission to contribute any reply to so simple a request for information is to us inexplicable.

The replies relating to "hospital luxuries" are also unsatisfactory, though one really good contribution has been received from "John."

We publish the remarks of the "Matron of a Fever Hospital," not because we in any way agree with them, but as a novel example of looking at the problems of the hospital commissariat.

### Hospital Luxuries.

AN EXTRA SHILLING PER HEAD PER WEEK.

With an extra allowance of 1s. per head on any given allowance many luxuries could be provided. The money might be expended all at once to give an exceptionally good meal once a week—on Sunday. Poultry, trifle, cheese, and fruit, for instance, in place of the usual cold joint, with tart or pudding. Or it might be used to give something extra with one meal on each day of the week. It would be sufficient to provide

EXTRAS.

Sunday.—Whipped cream with tart or pudding.
Monday.—Hot rolls and extra butter for breakfast.
Tuesday.—Buns for tea.
Wednesday.—Fruit in season after dinner.
Thursday.—Biscuits and extra butter for supper.
Friday.—Eggs (in the country, new-laid) for tea.
Saturday.—Fruit after dinner. "JOHN."

As matron of a hospital I have to provide the food for the staff (both nurses and domestics) out of the handsome sum of 9s. per head per week. This will be about £23 8s. per annum per head. Nurses and domestics all get the same food, which consists of fresh meat every day, bacon, eggs, fish, cheese, jam, Quaker oats, apples, tinned fruits, sausages, pies, milk puddings, bread, butter, etc. Now and again I am a shilling or two out of pocket, but this I attribute to the extravagance and waste of the domestics, or the 9s. ought to pay. However, I generally have my nurses as fit as any racehorse (their hours on duty commence from 6 A.M., with only two hours off duty every day), and when they leave me they invariably offer their services for any time I may have need of them, and this offer they carry out. Many of my nurses come from homes where the father has 35s. per week to keep himself, his wife, and, say, the nurse and one or two brothers. Out of this sum, after rent, rates, clothing, insurance, clubs, and boots, there cannot possibly be much more than 4s. 6d. per head per week left for food, yet many of these homes rear as strong and healthy children as many who cost five times as much. How is it, then, 4s. 6d. at home, 10s. in hospital? One remedy I can suggest, and that is to give the nurses extra wages and let them keep themselves. Find them house-room and firing, but let them provide their own food. Many of them would like it every bit as well; they would then get what they could best fancy and eat; there would not be so much waste. Where there is a large staff some might choose to join with another in provisions, but they would know less waste more pocket-money, and when they became a matron or held a responsible post much more economical and capable managers.—A MATRON OF A FEVER HOSPITAL.

### Editor's Notices.

**Contributions:**

Contributions should be written, or preferably typed, on one side of the paper only, and all articles sent in are accepted upon the distinct understanding that they are forwarded to THE HOSPITAL only.

The Editor cannot undertake to return MSS. not used, but when a stamped directed envelope is enclosed the MSS. may be returned if a special request is made.

Accepted articles and paragraphs of news will be paid for after publication at the scale rate.

**Address:**

To prevent delay all contributions and letters on editorial business must be addressed exclusively to the Editor, "The Hospital," 29 Southampton Street, Strand, London, W.C. It is important that this regulation shall be strictly observed.

**Correspondence:**

Correspondence on all subjects is invited. The name and address of correspondents must be given as a guarantee of good faith, but not necessarily for publication.

**Special Articles:**

Special articles are invited, and questions, inquiries, and paragraphs upon all matters relating to the work, administration, and management of general, special, mental, fever, cottage and convalescent hospitals, sanatoria, homes, institutions, societies and organisations for the treatment or care of the sick, injured and dependants of all classes. Every matter affecting the interests and welfare of the staff of all grades working in these institutions will receive special consideration.

**Administrative Medicine, Research and Items of News:**

Special terms will be paid for approved articles on subjects in this wide field by experts who have made some section of it their special study and interest. We wish to give prominence to every movement tending to advance accuracy of diagnosis, efficiency in treatment, and the development of modern methods to eradicate disease and promote the welfare of the suffering.

**A Bureau of Information:**

We invite inquiries and applications for information and help in providing for that numerous class of sufferers who require special care or house-room which they cannot obtain in their own homes or secure for themselves. We cannot, however, prescribe, or recommend practitioners.

**Books for Review:**

Publishers are particularly requested to send advance proofs of any new books of importance whenever possible, as the Editor has made arrangements to publish immediate reviews on a new plan.

**Photographs, Plans, Blocks, and Illustrations:**

It is requested that wherever possible MSS. may be accompanied by illustrations in any of the above forms. The name of the sender and of the article to which it belongs should be written on the back of each photograph, drawing, or block for purposes of identification.

**Local Papers:**

Newspapers containing reports or news paragraphs should be marked and addressed to the Sub-Editor.

**The Coupon System:**

A coupon will be found at the bottom of the third inside page of the cover each week. This coupon must be attached to every question or inquiry to which an answer is desired in THE HOSPITAL.

### The Workers' Section.

OUR purpose is to make this Supplement of THE HOSPITAL of the greatest possible service to every Institutional Worker. The term is employed in its widest sense, and includes all those individuals engaged in or associated with Charitable, Poor-Law, and Municipal Administration, as well as the Medical, Health, and Sanitary Services.

Although the section is primarily intended to help those seeking appointments, and numerous announcements of vacancies appear in its columns every week, it is felt there are also many other ways in which it can be of value. A number of pages have recently been added containing information which it is hoped will prove of assistance to many of our readers in their work.

It is our intention to make "The Institutional Worker" of practical utility to everyone connected with or attracted to Institutional life, and in this you can help us and yourself by showing a lively interest in this section and offering such suggestions for its improvement as may occur to you.

### Manager's Notices.

**Letters relating to the publication, sale, and advertising departments of "The Hospital" must be addressed to The Manager, "The Hospital," The Hospital Building, 28 and 29 Southampton Street, London, W.C.**

**Advertisements:**

To ensure insertion, all advertisements for THE HOSPITAL must reach the Manager not later than Wednesday morning in each week. For scale of charges see pages vii and 4.

**Special Rates** will be quoted for those institutions that agree to send all their vacancy, contract, and public notices to THE HOSPITAL each year, so as to make it a file of such announcements for ready reference.

**Subscriptions** may begin at any time, and are payable in advance. Cheques and Post-Office Orders should be crossed London County and Westminster Bank, Covent Garden Branch, and made payable to the Manager of THE HOSPITAL.

RATES OF SUBSCRIPTION (including Postage).

| *United Kingdom.* | | *Foreign and Colonial.* | |
|---|---|---|---|
| Three Months ... | ... 2s. 0d. | Three Months ... | ... 2s. 6d. |
| Six Months ... | ... 4s. 0d | Six Months ... | ... 5s. 0d. |
| Twelve Months ... | ... 6s. 6d. | Twelve Months ... | ... 8s. 8d. |

*SUBSCRIPTION FORM.*

*Please send me* THE HOSPITAL *for..............months, commencing with your issue of.............................., for which please find enclosed............................*

*Name*..................................................

*Address* ..................................................

..................................................

*Date*....................................

*To* ...................................... *Newsagent.*

**Remittances:**

**It is especially requested that remittances be made by Cheque or Postal Order, and in halfpenny stamps only when the amount is under sixpence.**

### The Scientific Press Publications

### IMPORTANT TO HOSPITAL SECRETARIES.

### ANALYSIS LEDGER.

**Specially Ruled in accordance with, and adapted to the requirements of, the New Index of Classification recently adopted by the King Edward's Hospital Fund for London, and which is now in operation.**

The Ledger is bound in half basil, green cloth sides, printed and ruled in best style on good paper, and is in every way a serviceable book for Hospital use.

**Price £1 12s. 6d.**

Secretaries requiring a copy of this Ledger should order at once to avoid delay.

### LEDGERS, ACCOUNT BOOKS, STOCK BOOKS of all kinds for large and small Institutions.

These are supplied already ruled and printed, or in accordance with the special requirements of institution managers.

### ANNUAL REPORTS AND PROSPECTUSES.

THE SCIENTIFIC PRESS are prepared to undertake the production of the Annual Reports and Prospectuses of every class of Institution, and to supply printed or typewritten copies of testimonials for the officials. Professional Cards.

### TEMPERATURE CHARTS.

MORNING, EVENING, FOUR-HOUR, MATERNITY.

| | | | | | |
|---|---|---|---|---|---|
| Size 10 inches by 8 inches | 12 | 3d. | 50 | 9d. | 250 3/3 |
| | 25 | 6d. | 100 | 1/4 | 500 6/6 |
| Postage extra. | | | | | 1,000 12/6 |

All Orders for 250 and over are sent Carriage Paid.

### CASE PAPERS

For Day and Night Nurse's Reports. Bound & unbound.

### DISTRICT NURSING ASSOCIATIONS'

Case, Visit, Time, and Loan and Account Books.

**THE SCIENTIFIC PRESS, LTD.,**
**28 and 29 Southampton Street, Strand, London, W.C.**

SEND FOR COMPLETE CATALOGUE.

### The Poor, Our Voluntary Hospitals and Nurses.

AN ADDRESS by The Right Rev.

THE LORD BISHOP OF LONDON.

Size $4\frac{1}{4} \times 5\frac{1}{2}$ in. Paper Cover. 3d. net; by post, 4d.

OF ALL BOOKSELLERS, OR

THE SCIENTIFIC PRESS, Ltd.,
28-29 Southampton Street, Strand, London, W.C.

### BOOKS FOR INSTITUTIONAL WORKERS.

A Legal Handbook for the Use of Hospital Authorities 2s. 6d.
The District Nursing Association: Hints on How to Start It . . . . . . . . . . 1s.
The Matron: Her Duties and Responsibilities . . 2s. 6d.
The Nurses' Pronouncing Dictionary . . . . . 2s.
Art of Feeding the Invalid . . . . . . . . 1s. 6d.
Furnishing and Appliances of a Cottage Hospital . 6d.
Burdett's Hospitals and Charities . . . . 10s. 6d.
Case-papers, Charts, Ledgers, Account Books, National Insurance pamphlets and every kind of Institutional literature.

Of all Booksellers, or of THE SCIENTIFIC PRESS, Ltd., 28 & 29 Southampton Street, Strand, London, W.C.

